# Skin cancers diagnosed at the dermatology department of a tertiary hospital in Jordan over one year

**DOI:** 10.1186/s43046-025-00268-0

**Published:** 2025-03-31

**Authors:** Jehad Shaher Alassaf, Laith Alabed, Salah Abdallat, Hamzeh Khair, Omar Ashokaibi, Hayat Khassawneh

**Affiliations:** 1 King Hussein Medical Center, Amman, Jordan; 2 Dermatology, King Hussein Medical Center, Royal Medical Services, Amman, Jordan; 3 Otorhinolaryngology, Royal Medical Services, Amman, Jordan; 4 Pathology, Princess Iman Center for Research and Laboratory Sciences, King Hussein Medical Center, Royal Medical Services, Amman, Jordan; 5 Dermatopathology, King Hussein Medical Center, Royal Medical Services, Amman, Jordan

**Keywords:** Skin cancer, Skin biopsy, Pathology, Basal cell carcinoma, Squamous cell carcinoma, Melanoma, Mycosis fungoides, Cutaneous T cell lymphoma

## Abstract

**Introduction:**

Skin tumours comprise an important fraction of dermatology practice. Skin tumours can be benign or malignant, and patients can present with a merely unsightly nodule to a rapidly growing nodule. The diagnosis is made on pathological basis, which is done after performing skin biopsies.

**Aim:**

In this study, we aim to describe the characteristics of malignant skin tumors diagnosed by skin biopsies over a one-year period (2023) at the Department of Dermatology, King Hussein Medical Center (KHMC).

**Methods:**

In this retrospective study, data from biopsies that were done at our department and diagnosed as skin cancers were collected, patients’ demographics were registered; including age, gender, and tumour location, and final diagnosis was recorded. Data was registered on an Excel® datasheet and analyzed using simple statistical methods.

**Results:**

There were 78 biopsies that were diagnosed as skin cancers at the department of Dermatology. Of these, the most common diagnosis was basal cell carcinoma with 38% of the biopsied cancers. Eighteen per cent were diagnosed as squamous cell carcinomas, and 15% were melanomas. Mycosis fungoides and cutaneous T cell lymphoma cases were included in this study and reached 18% of the diagnosed skin cancers in our patients.

## Introduction

Skin tumours are an important part of dermatology practice. They can be regarded as benign or malignant tumours. Malignant tumors can be further classified into non-melanoma skin cancer (NMSC) and melanoma skin cancer. The most common NMSC is the broadly termed keratinocytic tumors, which include basal cell carcinoma and squamous cell carcinoma.

The exact prevalence of skin cancer is likely underestimated, but is increasing in the past few decades [1].

Head and neck area is a common location for skin cancer development, since ultraviolet light exposure is an important etiologic factor for both melanoma and non-melanoma skin cancers [1].

Skin biopsy is central to establishing a diagnosis in cutaneous tumours. It is a common procedure that is done for diagnostic and therapeutic purposes [2].

## Aim

In this study, we aim to describe the characteristics of the patients of malignant skin tumors diagnosed by skin biopsies over a one-year period at the Department of Dermatology, King Hussein Medical Center, Royal Medical Services.

## Methods

In this retrospective study, data from biopsies that were done in the year 2023 (from 1/1/2023 till 31/12/2023) at our department and diagnosed as skin cancers was collected from the dermatology department registries. The patients enrolled were those whose diagnostic biopsies were done at the department of dermatology and whose biopsies were diagnosed as skin cancer after pathological examination. No other samples (i.e. referrals to plastic surgery or samples not diagnosed as skin cancers) were included. Patients’ demographics were registered; including age, gender, tumour location, and the final diagnosis. Data was collected for one year from the period 1/1/2023 till 31/12/2023 and was analyzed.

The institutional review board of the Royal Medical Services and ethical committee acceptance was obtained.

## Results

A total of 78 of the biopsy specimen taken at the dermatology department in the year 2023 were pathologically diagnosed with skin cancer. All the patients with a suspected clinical diagnosis of skin cancer were biopsied.

**Fig. 1:**
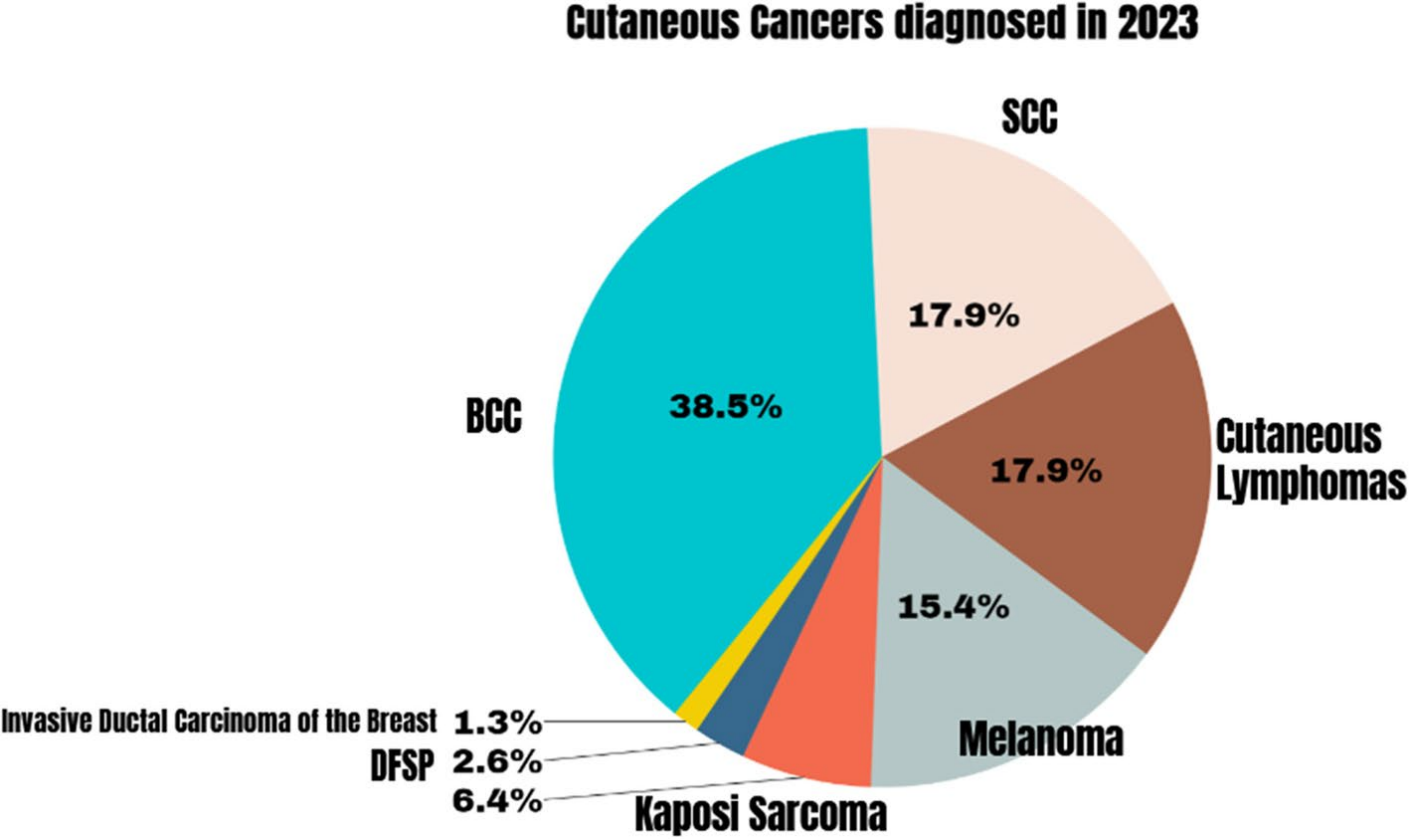
Distribution and percentages of cutaneous cancers diagnosed at King Hussein Medical Center dermatology department in 2023

Of these, 30 biopsy specimens (38%) were diagnosed with basal cell carcinoma, including 2 basosquamous carcinomas. Fourteen biopsy specimens (18%) were diagnosed as squamous cell carcinomas, and 12 specimens were melanomas (15%). Mycosis fungoides and cutaneous T cell lymphoma cases were included in this study and were 14 specimens (18%). A graphic representation of the results is shown in (Fig. 1).

### Basal cell carcinoma (BCC)

**Fig. 2:**
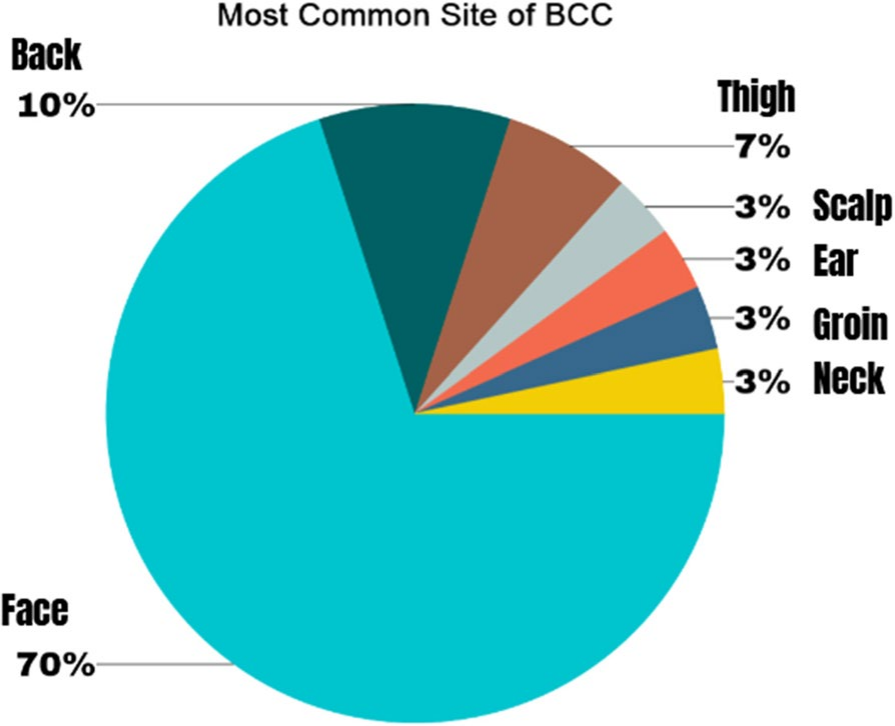
Sites of basal cell carcinomas (BCCs) diagnosed at King Hussein Medical Center dermatology department in 2023

Basal cell carcinomas were the most common skin cancer in our patients, comprising 38% of the cancers diagnosed, including two basosquamous carcinomas. A total of 30 of the specimens taken from 27 patients were diagnosed with basal cell carcinomas (38%). One patient had three different basal cell carcinomas, and one had two. Only one biopsy was taken from each tumour and patients were sent to plastic surgery for complete excision if needed. Of the 27 patients, 14 were males, comprising 52% of the patients diagnosed with basal cell carcinoma, and 13 were females, comprising 48% of the patients diagnosed with basal cell carcinoma. The age range in male patients was 41 – 84 years, with a mean age of 63.14 years, while the age range in females was 50 – 81 years, with a mean age of 66 years. The most common site of BCCs diagnosed was the face, with 21 out of the 30 cases presenting on the face. Other sites involved were the back (3 cases), thigh (2 cases), neck (1 case), groin (1 case), scalp (1 case), and ear (1 case). Totally, the head and neck area was involved in 23 out of the 30 cases (77%). See (Fig. 2).

### Squamous cell carcinoma (SCC)

Squamous cell carcinoma was the second most common cancer diagnosed in our patients; with 14 specimen diagnosed in 14 patients, comprising 18% of the specimens. SCC was diagnosed in 7 males and in 7 females; with an age range of 52 – 84 years in male patients and a mean age of 67.9%, and an age range of 41 – 85 years in female patients and a mean age of 64.7 years, and a total mean age of 66.3%.

**Fig. 3:**
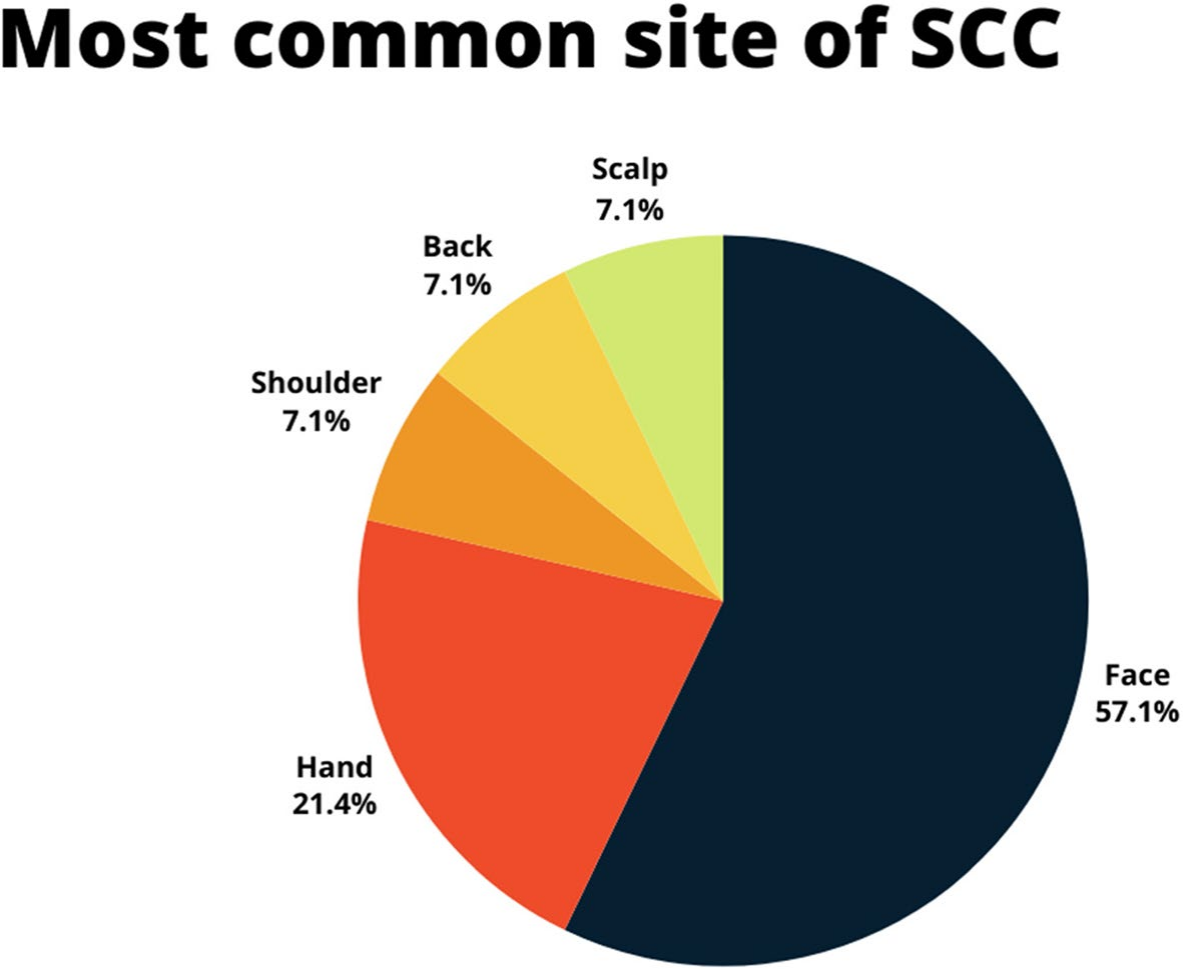
Sites of squamous cell carcinomas (SCCs) diagnosed at King Hussein Medical Center dermatology department in 2023

The most common site of the SCCs in our patients was the face with 8 out of 14 specimens (57%), followed by the hand in 3 out of 14. Less common sites include the shoulder, back, and scalp, with one case each (1/14). See (Fig. 3).

### Cutaneous lymphomas

Cutaneous lymphoma was diagnosed in 14 patients, with 13 cases of those diagnosed with mycosis fungoides and one case diagnosed with follicular B cell lymphoma.

Nine of the 14 patients with cutaneous lymphoma were males (64%) with an age range of 12–85 years and a median age of 52, while 5 of the 14 patients were females, with an age range of 17–73 years and a mean age of 48.6, and a total mean age of 48.6 years. The case of follicular B cell lymphoma was diagnosed in a 73-year-old female patient.

### Melanoma

A total of 12 biopsies in 12 patients were diagnosed as melanoma. The patients were 6 males and 6 females, with an age range of 31–74 in the males and a mean of 60.5 years, and an age range of 310–85 in the females with a mean of 66.5 years. The total mean age was 63.5 years.

**Fig. 4:**
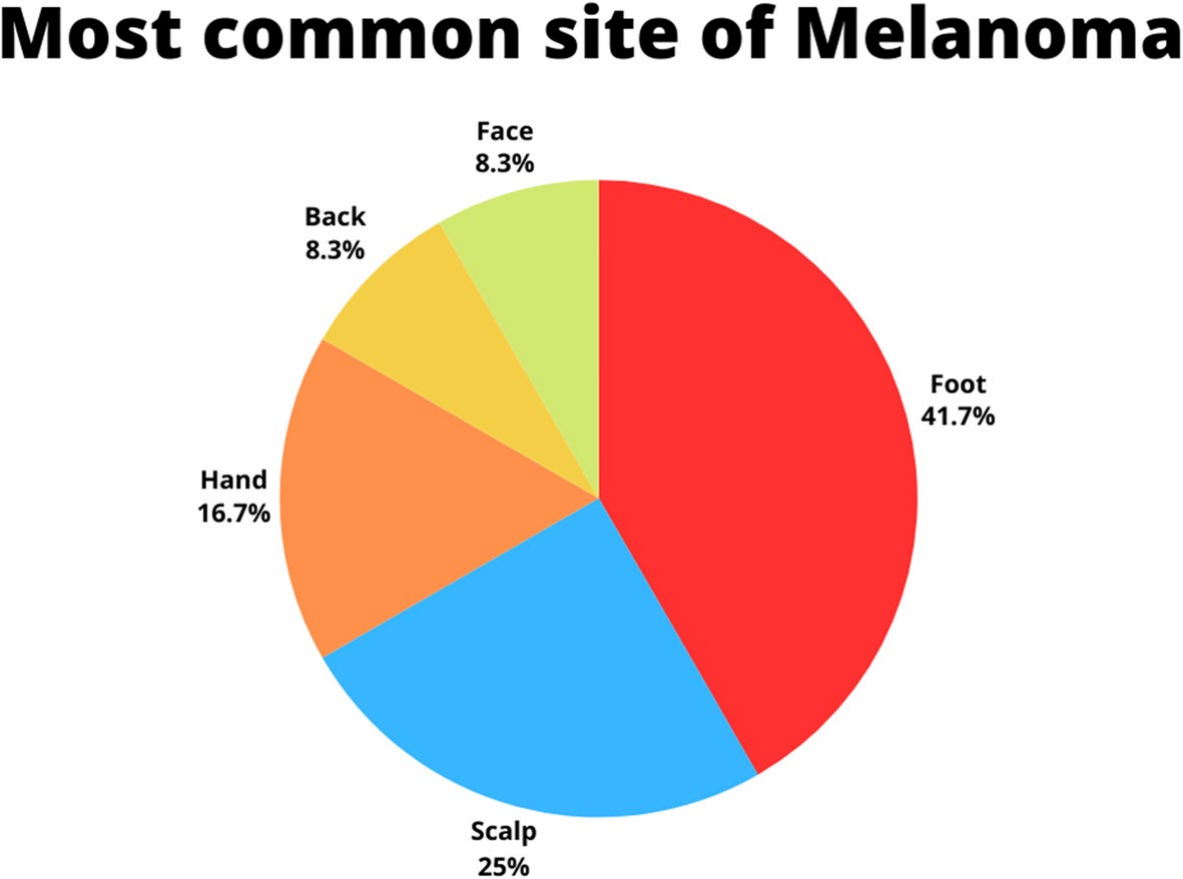
Sites of melanomas diagnosed at King Hussein Medical Center dermatology department in 2023

Most common sites in the melanomas diagnosed include the foot (5/12 patients), followed by the scalp (3/12 patients), then the hand (2/12 patients). Face and back locations comprised one case each (1/12). See (Fig. 4).

### Kaposi sarcoma

There were a total of 5 Kaposi sarcoma biopsies in 4 patients. These were diagnosed in three male and one female patients. The ages of the patients were 71, 68, 66, & 57 years.

As for the location, four of the five biopsies done were from the lower limbs, while one biopsy was taken from the upper limb.

### Miscellaneous

Dermatofibrosarcoma protuberans was the diagnosis in 2 patients. Both were females, 14 and 28 years of age with a mean age of 21 years. As for the location, one biopsy was from the arm and one was from the back.

Invasive ductal carcinoma of the breast was diagnosed in one patient. She was a 75-year-old female.

**Fig. 5:**
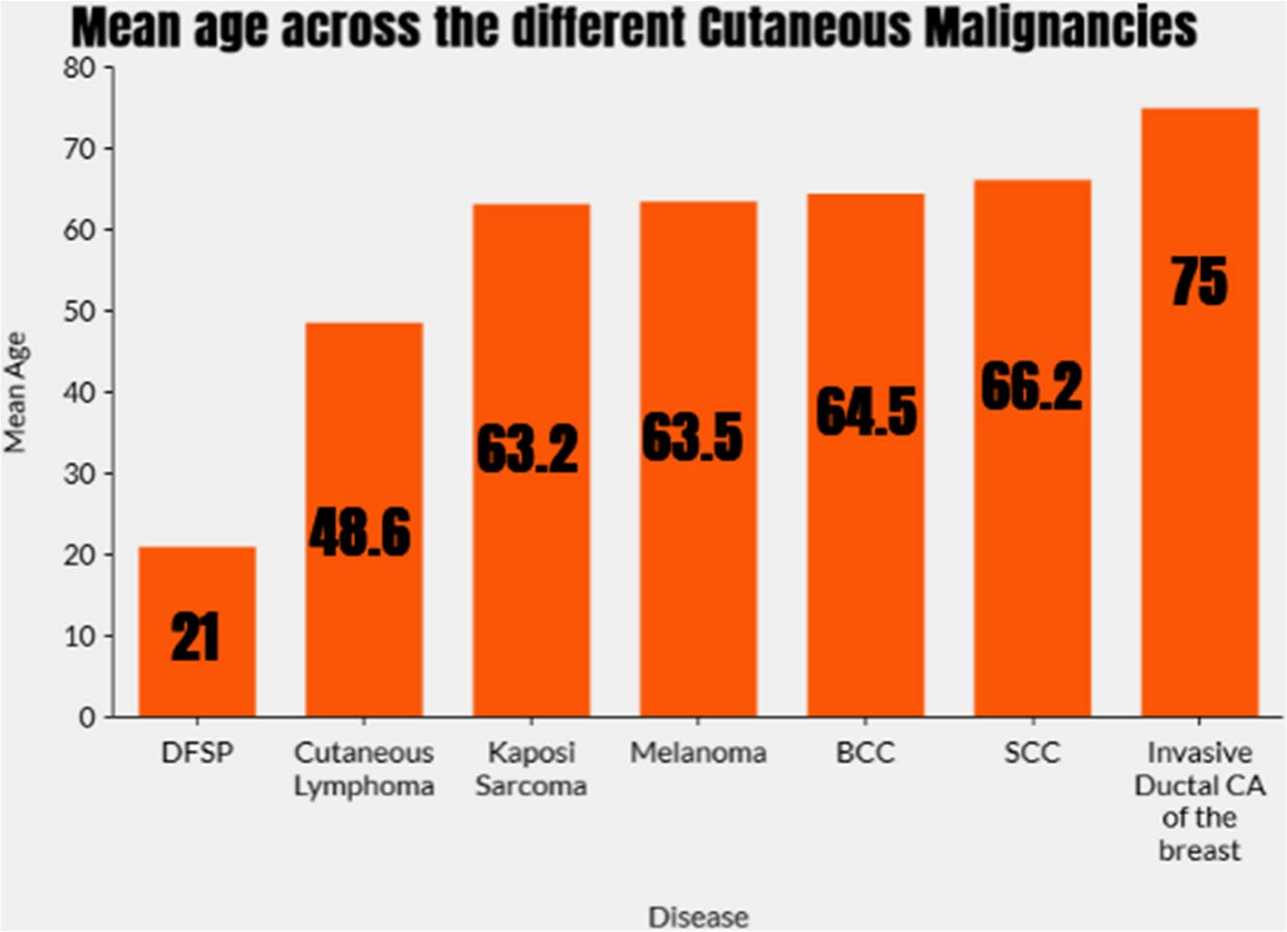
Mean age of patients at diagnosis of different malignancies diagnosed at King Hussein Medical Center dermatology department in 2023

Figure 5 shows the mean age at diagnosis of different cutaneous malignancies in the patients of this study.

## Discussion

Skin cancers are a very important part of the dermatology practice. They can be broadly categorized into melanoma and non-melanoma skin cancers [1].

A great number of skin cancers is thought to be underreported. Nonetheless, skin cancer prevalence is higher than the prevalence of all other malignancies [1, 3]. Non-melanoma skin cancer is the most common malignancy overall, and the most common cutaneous malignancy; comprising 90% of skin cancers. Basal cell carcinoma is the most common skin cancer, comprising 70% of non-melanoma skin cancers, and the most common malignancy overall [4].

The incidence of skin cancers is increasing worldwide [5]. Between 2007 and 2017, the incidence has increased by 33% [6] and is expected to increase.

Jordan is a country in the Middle East located in the area roughly between 29° North and 33° North latitude, and 35° East to 39° East longitude [7]. The climate in Jordan is Mediterranean in the west and arid in the south and east, with a generally warm climate, cold winters, a UV index of 5–7 in winter and 8–10 in summer, and an average 310 days of sunshine a year [8].

The current population of Jordan is around 11.6 million people with the majority of Arabs (94%), including those of Jordanian and of Palestinian origins, Bedouin Arabs, and smaller minorities of Circassians and Chechens, Armenians, Kurds, and the recent immigrant Syrians [9]. Collectively, the major ethnicity in Jordan is Arabs. The most prevalent Fitzpatrick skin type is likely III and IV as the middle eastern skin is generally olive type and tans easily. Other Fitzpatrick skin types are also seen. The age distribution of the population of Jordan reflects a predominance of the young population, with a third of the population below 15 years of age and 63% of the population below 29 years of age. People who are 60 years old and above constitute 5.4% of Jordan’s population [10].

Of note, the total number of dermatology clinic visits at KHMC in 2023 for various reasons and diseases were around 15,300 visits, with an estimated 7000 patients after exclusion of recurrent visits. A total of 683 skin biopsies were performed at the dermatology department at KHMC in the year 2023 for various reasons. Of these, inflammatory conditions comprised the majority of reasons for which skin biopsies were performed, with 517 biopsies, while 166 skin biopsies were performed to rule out malignancies. 78 skin biopsies resulted in the diagnosis of various skin cancers.

Our data shows that the most commonly diagnosed malignant skin cancer in our patients over the year was BCC, which is consistent with the literature being the most common cutaneous malignancy [6]. The literature shows that worldwide BCCs are the most common cancer in the white population and SCC being the most common in black ethnic groups [11, 12]. Our study shows BCCs to be more common in our patients of middle eastern origin. This is also supported by the numbers from the national registry in a published article [13] but also needs a wider look at our numbers in the future.

The most common location of BCCs in our patients was shown to be the head and neck with 77% of the cases, which is also the most common location of BCC worldwide [14] with BCCs on the face comprising 70% of the cases (21/30), and the rest in the scalp and ear with 1/30 cases, or 3% each. This relates to UV light exposure, which is the major etiopathogenic factor in BCCs.

BCCs in our patients also were slightly more common in males and affected males at an earlier age than females, which is consistent with results obtained in Jordan [15].

The second most commonly diagnosed malignant skin tumour in our data was squamous cell carcinoma, accounting for 18% of the cancers. Worldwide, SCCs are also the second common skin cancer [11], while in people of colour or of black ethnic background it is the most common skin cancer [12]. Our numbers reflect the worldwide numbers in our middle eastern population but larger cohorts are required.

Mycosis fungoides is the most common type of cutaneous lymphomas [16], reaching 93% in our cases. Cutaneous lymphomas were diagnosed in males more than in females (1.8:1) with a mycosis fungoides male:female ratio of (2.25:1), which is also consistent with the literature but with a slightly higher male: female ratio than international numbers, which range from 1.4:1 to 1.7:1 [16–18]. As for the age at diagnosis, the mean age at diagnosis of patients form our cohort was 48.6 years, which is less than international numbers, which range from 51.3 to 59.2 [18].

As for melanomas, a significant number of melanoma cases was diagnosed. Importantly, the melanomas diagnosed in our patient show predilection for acral sites (foot then hand), which is consistent with the clinical subtype of acral melanomas, which are the most common melanoma type in people of color and non-white ethnicities [19, 20].

Kaposi sarcoma and dermatofibrosarcoma protuberans cases diagnosed during 2023 at our department were too few to draw significant conclusions. All of the four patients had normal white cell count, and all had comorbidities. Three patients were males, and three patients were on chemotherapy at the time of presentation [21].

## Limitations

One limitation of this study is that some skin cancers get therapeutic excisional biopsies at the plastic surgery outpatient day case department, so our numbers may be an underrepresentation of the real situation. Yet, they reflect our practices. Another limitation is that not all skin cancers get biopsied, especially early basal cell carcinoma and this may also reflect in numbers.

Our data shows 78 skin cancers diagnosed over one year. The skin biopsy procedures done at the dermatology department at KHMC for various reasons in 2023 was 683. One hundred sixty-six of these were done to rule out malignancies. This shows that a great proportion of our workload is inflammatory and other non-cancerous dermatoses.

The case numbers in our study were obtained over a year’s period. This could give a glimpse but a larger study is required for better demographics and analysis.

## Conclusion

Skin biopsy and pathologic examination is essential in diagnosing skin malignancies. It is of interest to have information on the skin biopsies done at a tertiary hospital in Jordan, to get some demographic data on skin cancers in our population. This can be expanded in the future, to get more accurate data and to monitor shifts or trends [22–26].

## Data Availability

Data and material used in this study is available from the authors upon request and upon approval.
